# Divergent PML and Nucleolar Body Programs Distinguish Protected from Vulnerable Neurons in FTLD-TDP

**DOI:** 10.21203/rs.3.rs-11089898/v1

**Published:** 2026-09-30

**Authors:** Oseas Medina, Alissa Nana Li, Lily Talley, Stephanie E. Gaus, Joel H. Kramer, Jennifer S. Yokoyama, Howard J. Rosen, Maria Luisa Gorno Tempini, Salvatore Spina, Lea T. Grinberg, Bruce L. Miller, William W. Seeley

**Affiliations:** University of California; University of California; University of California; University of California; University of California; University of California; University of California; University of California; University of California; University of California; University of California; University of California

**Keywords:** TDP-43, frontotemporal lobar degeneration, PML nuclear bodies, SUMO1, nucleolus, protein quality control

## Abstract

TDP-43 nuclear depletion and cytoplasmic aggregation are hallmarks of frontotemporal lobar degeneration (FTLD) and motor neuron disease, yet the events preceding depletion remain poorly characterized. PML nuclear bodies and the nucleolus are phase-separated protein quality control (PQC) compartments; SUMO1 is integral to PML nuclear body assembly and accumulates in the nucleolus under proteostatic stress. How they remodel in relation to TDP-43 pathobiology has not been examined at single-cell resolution in human tissue. We analyzed 18,742 anterior cingulate cortex neurons across 29 subjects (10 sporadic FTLD-TDP, 10 C9orf72-associated FTLD-TDP, 9 controls) by multichannel confocal immunofluorescence, scoring each neuron for five nuclear body phenomena and TDP-43 nuclear depletion. Three phenomena were elevated in nuclear TDP-43-preserved patient neurons relative to controls and returned to approximately control levels in depleted neurons, consistent with a protective role (control → preserved → depleted): SUMO1 nucleolar granules (3.2% → 13.9% → 5.0%), SUMO1 nucleolar puncta (0.2% → 11.0% → 3.1%), and PML nucleolar contacts (2.5% → 6.4% → 1.7%). In contrast, PML nuclear membrane contacts (0.1% → 2.6% → 19.9%) and macro-PML bodies (0.0% → 0.9% → 15.6%) increased progressively, and both were associated with depletion (odds ratio 9.2, 95% CI 6.1–14.0 and 20.2, 95% CI 13.9–29.4), consistently across sporadic and C9orf72-associated disease. Within nuclear TDP-43-preserved neurons, protection-associated phenomena rarely co-occurred with PML nuclear membrane contacts (odds ratio 0.39–0.54, all p < 0.05) but co-occurred with each other (odds ratio 3.3–4.0), defining two segregated remodeling programs; depletion fell from 15.3% in neurons bearing no protection-associated phenomenon to 2.3% in those bearing all three (Kendall’s τ = −0.135). These findings identify opposing nuclear body remodeling programs in FTLD-TDP, expand the spectrum of pre-depletion changes, implicate phase-separated nuclear PQC compartments as determinants of neuronal fate, and raise the possibility that SUMO1 nucleolar accumulation is a general marker of neuronal stress.

## Introduction

Cytoplasmic aggregation and nuclear depletion of TDP-43 is a defining pathological feature of frontotemporal lobar degeneration with TDP-43 inclusions (FTLD-TDP) and amyotrophic lateral sclerosis (ALS-TDP) [5,31,33,34,41]. Nuclear TDP-43 loss leads to widespread RNA processing disruption, including aberrant inclusion of cryptic exons in essential neuronal transcripts [11,32,44]. Despite the centrality of TDP-43 depletion to disease pathogenesis, the cellular events that precede depletion and that determine which neurons are vulnerable to TDP-43 pathobiology remain poorly understood. The inventory of established pre-depletion changes is limited, and whether individual changes represent protective/compensatory responses or early pathobiological events has been difficult to resolve.

The cell nucleus contains several classes of phase-separated condensates that compartmentalize essential functions [1,2,6,20]. Among these condensates, promyelocytic leukemia (PML) nuclear bodies and the nucleolus are of particular relevance to neurodegenerative proteinopathies, and both have been described as phase-separated compartments that concentrate protein quality-control factors and misfolded clients [39]. PML nuclear bodies are membraneless organelles assembled through a network of SUMO–SUMO-interacting motif (SIM) interactions that create a scaffold for client protein recruitment [7,28,47]. SUMO1, a constitutive component of PML nuclear bodies, also accumulates in the nucleolus under proteotoxic stress as part of the nucleolar protein quality-control response [36]. PML bodies function as nuclear PQC compartments: under proteostatic stress, they sequester misfolded proteins including defective ribosomal products and coordinate their processing through SUMOylation-ubiquitination cascades coupled to the p97/VCP disaggregase [17,19,22,27,38,45]. The nucleolus, a phase-separated structure organized to enable ribosomal RNA transcription, has similarly emerged as a stress-responsive compartment that sequesters misfolded and aggregation-prone proteins under conditions of proteotoxic challenge [9,10,16,29]. Notably, SUMO-1 conjugates specifically redistribute from nuclear dots to nucleolar structures upon proteasome inhibition, where they coordinate with the ubiquitin-proteasome system to maintain nucleolar integrity [36]. This nucleolar SUMO-1 response is paralog-specific, distinct from SUMO-2/3 behavior, and targets substrates including nucleophosmin/B23, histones, and ribosomal proteins [36].

Recent work has begun to connect these phase-separated PQC compartments to TDP-43 biology. SUMOylation of TDP-43 maintains its nuclear solubility by promoting recruitment to PML bodies, where ubiquitin-dependent quality control machinery processes misfolded species [19,22,27,38,45]. Loss of PML nuclear bodies has been observed in cellular models of ALS-FTD, including patient *C9orf72*-expansion iPSC-derived motor neurons and non-neuronal cells expressing the expanded GGGGCC repeat [4,14,15,53]. Alterations in PML body number and morphology have been reported in familial ALS-FTD tissue. In these settings, the loss of nuclear PML was reported to impair compartmentalization of defective ribosomal products, driving their accumulation in cytoplasmic stress granules and implicating a breakdown of nuclear–cytoplasmic quality-control cross-talk [4]. However, the landscape of nuclear body remodeling in FTLD/ALS-TDP and specifically how different configurations of PML bodies and SUMO-modified structures relate to TDP-43 status at single-cell resolution has not been systematically examined.

Here, we leverage multichannel confocal immunofluorescence to characterize nuclear body remodeling in 18,742 anterior cingulate cortex neurons from participants with sporadic FTLD-TDP, *C9orf72*-associated FTLD-TDP, and neurologically normal controls. We identify two opposing remodeling programs within phase-separated nuclear compartments: a potentially protective SUMO1/PML nucleolar pattern associated with nuclear TDP-43 retention and a TDP-43 aggregate-linked PML redistribution to the nuclear envelope that may mark pathobiology that precedes or accompanies vulnerability. Two independent analytical approaches, cross-sectional prevalence across disease states and single-cell co-occurrence, converge on this classification. These findings implicate phase-separated nuclear PQC as a potential driver of neuronal fate in FTLD-TDP, substantially expand the known repertoire of pre-TDP-43-depletion pathological changes, and raise the possibility that nucleolar activation/recruitment SUMO1/PML represents a protective response to neuronal stress [36] with potential as a therapeutic target.

## Methods

### Human brain tissue

Brain tissue was obtained from the UCSF Neurodegenerative Disease Brain Bank (NDBB). The study cohort comprised 29 participants: 20 with autopsy-confirmed FTLD-TDP, type B/U, including 10 with sporadic disease (sFTD) and 10 with *C9orf72* repeat expansion-associated illness (C9-FTD); and 9 neurologically normal controls (Table 1). Clinical presentations comprised bvFTD, FTD-ALS, nfvPPA-ALS and svPPA. Cases were matched for sex and postmortem interval (PMI); controls were older than both patient groups (Table 1). Exclusion criteria included high Alzheimer disease neuropathologic change (ADNC), diffuse neocortical Lewy body disease, significant cerebrovascular pathology, PMI > 48 hours, and neuropsychiatric illness in controls. Participants (or their surrogates) provided informed consent to undergo autopsy, in keeping with the guidelines outlined in the Declaration of Helsinki. All procedures were approved by the UCSF Committee on Human Research.

### Immunofluorescence

Formalin-fixed, paraffin-embedded sections of anterior cingulate cortex (ACC) were cut at 6 μm thickness. Antigen retrieval was performed in citrate buffer. Multichannel immunofluorescence staining was performed using antibodies against NeuN to identify neurons, pan-TDP-43 to identify nuclear TDP-43 levels and pathological inclusions, SUMO1, PML, and B23 to identify the nucleolus (Online Resource 1). Species cross-reactivity was managed through direct fluorophore conjugation of one antibody within any pair of antibodies raised against the same species. All protocols were optimized in-house and validated with positive and negative controls.

### Confocal microscopy

Images were acquired on a Leica SP8 confocal laser scanning microscope. Imaging used an HC PL APO CS2 63×/1.40 oil-immersion objective (numerical aperture 1.40) at a pixel size of 0.18 μm and a pinhole of 1.0 Airy unit, with 600 Hz unidirectional scanning and no frame or line averaging. The five fluorophores were recorded by sequential (between-frame) scanning to minimize spectral bleed-through, using a 405 nm diode laser and a white-light laser tuned to 499, 553, 598, and 653 nm. Emission was collected on hybrid (HyD) and PMT detectors over the following windows: CoraLite488 (TDP-43), 504–558 nm at 499 nm excitation; the anti-mouse B23 secondary, 420–452 nm at 405 nm; AlexaFluor555 (SUMO1), 558–603 nm at 553 nm; the anti-rabbit PML secondary, 610–655 nm at 598 nm; and the far-red anti-guinea pig NeuN secondary, 658–775 nm at 653 nm. Antibody clones, catalog numbers, dilutions, and channel assignments are provided in Online Resource 1. Single-plane two-dimensional images were acquired for each region of interest, with the focal plane optimized to capture nuclear features. Regions of interest were placed over cortical layers III–VI to sample the pyramidal projection neuron population most affected in FTLD-TDP, avoiding layer I and, where the laminar boundary was distinct, layer II; laminar boundaries were not always unambiguous, and layer identity was not recorded for each region. To ensure adequate representation of the relatively infrequent nuclear TDP-43-depleted neurons, regions were also selected to include fields with higher nuclear TDP-43 depletion density, assessed at the field level during region selection. Region selection was performed before blinding; sections were then relabeled by a second investigator (A.N.L.) to blind the rater to participant identity and diagnosis, and all SUMO1 and PML phenomena were subsequently scored on the pre-drawn regions while blinded to nuclear TDP-43 status. A minimum of 550 neurons per subject were imaged across two tissue sections. Acquisition parameters (laser power, pixel dwell time, detector gain) were standardized across all subjects.

### Nuclear remodeling phenomenon evaluation

Pilot studies using the immunofluorescence staining procedures outlined above suggested at least five distinct nuclear body phenomena related to SUMO1 and PML (Fig. 1). Each neuron was scored for the presence or absence of these features, as binary calls. Scoring was performed by an expert rater blinded to participant identity and diagnostic group. To avoid unblinding by the presence of TDP-43 pathobiology, the SUMO1/PML channels were evaluated prior to TDP-43 assessment. Phenomena were defined as follows: (1) SUMO1 nucleolar granules: SUMO1 distributed throughout the nucleolus (pan-nucleolar) as fine granules, colocalizing with B23; (2) SUMO1 nucleolar puncta: one or more discrete SUMO1-positive foci within the B23-positive nucleolus, rather than a pan-nucleolar distribution; (3) PML nucleolar contacts: a PML nuclear body in direct contact with or overlapping the B23-positive nucleolus; (4) PML nuclear membrane contacts: a neuron was scored positive when most of its PML nuclear bodies (semi-quantitatively, ~ 50% or more) abutted or overlapped the nuclear envelope delineated by SUMO1; (5) Macro-PML bodies: a PML nuclear body enlarged such that approximately three normal-sized PML bodies could fit within it. TDP-43 nuclear depletion was noted, with or without an accompanying cytoplasmic inclusion, when marked reduction or absence of nuclear TDP-43 signal relative to surrounding neurons within the same field was observed. B23/nucleophosmin, a constitutive nucleolar protein, was used to delineate the nucleolus. Neurons were classified by nuclear TDP-43 status as nuclear TDP-43-depleted (marked reduction or loss of nuclear TDP-43 immunoreactivity, with or without a cytoplasmic inclusion) or nuclear TDP-43-preserved (nuclear TDP-43 immunoreactivity retained).

### Statistical analysis

#### Cross-sectional prevalence analysis.

To classify phenomena as potentially protection-associated vs. vulnerability-associated, we compared prevalence across three neuronal populations: control neurons (all nuclear TDP-43-preserved), patient nuclear TDP-43-preserved neurons, and patient nuclear TDP-43-depleted neurons. Prevalence was calculated as the percentage of neurons counted within each population in the sampled fields, not of the original neuronal population. Phenomena showing inverted-U trajectories (increased in nuclear TDP-43-preserved patient neurons relative to controls and returned to approximately control levels, not reduced below controls, in nuclear TDP-43-depleted patient neurons) were classified as protection-associated; those showing progressive increases across the three populations were classified as vulnerability-associated. These classifications were not intended to imply definitive roles but rather to nominate features for evaluation in mechanistic studies.

#### Association with TDP-43 depletion.

For each phenomenon, the odds of nuclear TDP-43 depletion in phenomenon-positive versus phenomenon-negative neurons were estimated by logistic regression across all disease neurons. Odds ratios and 95% confidence intervals were obtained with subject-clustered (sandwich) standard errors to account for the nesting of neurons within subjects, and were confirmed with a random-intercept mixed model.

#### Co-occurrence analysis.

Within patient nuclear TDP-43-preserved neurons, we tested whether protection-associated and vulnerability-associated phenomena co-occur at the single-cell level using Fisher’s exact test with Haldane-corrected odds ratios and 95% confidence intervals. A protection-associated repertoire score (0–3) was computed as the sum of the three protection-associated phenomena within each neuron. Association between protection-associated repertoire and nuclear TDP-43 depletion was assessed using Kendall’s τ.

#### Pairwise phenomenon associations.

All pairwise associations among the five phenomena were tested using Fisher’s exact test with Haldane-corrected odds ratios in disease nuclear TDP-43-preserved neurons, those in which both protection-associated and vulnerability-associated phenomena were detected at testable frequencies. Results were visualized using an UpSet plot and correlation heatmap.

#### Genotype interaction analysis.

Genotype effects were assessed with a phenomenon × *C9orf72*-status interaction term in per-neuron logistic models. Because genotype is a subject-level exposure, each interaction was estimated under three specifications: logistic regression with subject-clustered standard errors, generalized estimating equations with an exchangeable working correlation, and a random-intercept mixed model. An interaction was considered robust only if it was concordant across all three specifications, stable under leave-one-subject-out resampling, and significant after Benjamini–Hochberg correction across the five phenomena.

All analyses were performed in Python 3.12 using pandas, scipy, and numpy. Statistical significance was set at p < 0.05 (two-tailed). No corrections for multiple comparisons were applied to the primary prevalence and co-occurrence analyses, given their independent and hypothesis-driven nature; all effect sizes and confidence intervals are reported to allow independent assessment.

## Results

### Cohort and nuclear remodeling phenomenon distribution

We analyzed 18,742 neurons from 29 subjects across 58 images (Table 1). Patients contributed 13,276 neurons (6,858 C9-FTD; 6,418 sFTD), of which 11,593 (87.3%) retained nuclear TDP-43 and 1,683 (12.7%) were nuclear TDP-43-depleted. Controls contributed 5,466 neurons, none of which showed TDP-43 depletion. TDP-43 depletion rates were comparable between sFTD (13.1%) and C9-FTD (12.3%). Among the five nuclear body phenomena, SUMO1 nucleolar granules were the most prevalent in patient nuclear TDP-43-preserved neurons (13.9%), followed by SUMO1 nucleolar puncta (11.0%), PML nucleolar contacts (6.4%), PML nuclear membrane contacts (2.6%), and macro-PML bodies (0.9%).

### Cross-sectional prevalence analysis identifies protection-associated and vulnerability-associated phenomena

The five disease-associated nuclear remodeling phenomena we observed fell into two hypothesized pseudo-temporal sequences. Putatively “protective” phenomena peaked in patient neurons with preserved nuclear TDP-43, whereas “vulnerability-associated” phenomena increased progressively from controls to patient nuclear TDP-43-preserved neurons to patient nuclear TDP-43-depleted neurons (Fig. 2a).

Three phenomena followed protection-associated inverted-U trajectories (Fig. 2b). SUMO1 granules increased from 3.2% in controls to 13.9% in patient nuclear TDP-43-preserved neurons (4.3-fold enrichment) before falling to 5.0% in nuclear TDP-43-depleted neurons. SUMO1 puncta showed the most dramatic trajectory, rising from 0.2% in controls to 11.0% in patient nuclear TDP-43-preserved (55-fold enrichment) before declining to 3.1% in nuclear TDP-43-depleted neurons. PML nucleolar contacts increased from 2.5% to 6.4% (2.6-fold) before dropping to 1.7%. These inverted-U trajectories are consistent with compensatory protective responses that are mounted in disease and either stave off TDP-43 mislocalization or can no longer be mounted once TDP-43 nuclear integrity fails.

In contrast, PML nuclear membrane contacts increased progressively across the three populations: 0.1% in controls, 2.6% in disease nuclear TDP-43-preserved, and 19.9% in disease nuclear TDP-43-depleted neurons, a 199-fold increase from controls to nuclear TDP-43-depleted neurons. This progressive escalation is consistent with a pathological change that accumulates prior to TDP-43 depletion and either persists or continues to emerge in neurons lacking nuclear TDP-43. Macro-PML bodies also increased in a step-wise manner across the three neuronal populations (Fig. 2c). They were absent in controls (0 of 5,466 neurons) and rare in disease nuclear TDP-43-preserved neurons (105/11,593, 0.9%) but rose sharply in nuclear TDP-43-depleted neurons, to 15.6% of neurons pooled across cases (14.1% as a per-subject mean), a progressive trajectory that parallels PML nuclear membrane contacts. The association with TDP-43 depletion was stronger for macro-PML bodies than for membrane contacts (OR = 20.2, 95% CI 13.9–29.4 vs. OR = 9.2, 95% CI 6.1–14.0) (Fig. 3a).

Rather than representing separate processes, the two vulnerability-associated phenomena appear to mark successive points along a single envelope-associated trajectory. Membrane contacts were identified as any PML body apposed to the nuclear envelope, whereas Macro-PML bodies were based on enlargement; most Macro-PML bodies were themselves membrane-associated (70.8% overall; 66.7% in nuclear TDP-43-preserved and 72.5% in nuclear TDP-43-depleted neurons), and the two co-occurred far more often than expected within nuclear TDP-43-preserved neurons (OR = 95.8, p < 10^− 87^). These observations are consistent with recruitment of PML bodies to the nuclear envelope followed by their enlargement, with the larger, more TDP-43 depletion-associated macro-PML state potentially marking a more advanced stage of the same process.

### Protection-associated and vulnerability-associated phenomena were strongly segregated among individual neurons

If protection-associated and vulnerability-associated phenomena reflect distinct cellular programs, they should segregate into different neurons. We tested this hypothesis by examining co-occurrence patterns within patient neurons with preserved nuclear TDP-43 (Fig. 3).

All three protection-associated phenomena, when present, lowered the likelihood that PML nuclear membrane contacts were also present in the same cells: SUMO1 granules (OR = 0.54, 95% CI: 0.42–0.71, p < 0.001), SUMO1 puncta (OR = 0.41, 95% CI: 0.22–0.76, p = 0.005), and PML nucleolar contacts (OR = 0.39, 95% CI: 0.18–0.83, p = 0.014). Macro-PML bodies showed point estimates in the same direction (OR = 0.24–0.31 across the three protection-associated phenomena), but these comparisons were underpowered: Macro-PML bodies were rare in nuclear TDP-43-preserved neurons, leaving only two to five neurons that carried a protection-associated phenomenon together with a Macro-PML body, and none of the three reached significance after accounting for subject clustering (p = 0.05–0.10). Because Macro-PML bodies almost always co-occurred with PML nuclear membrane contacts (OR = 95.8), however, the robust segregation of the protection-associated phenomena from membrane contacts implies their segregation from the vulnerability-associated program as a whole. Therefore, the protection-associated nucleolar response was found in different neurons than those exhibiting the vulnerability-associated nuclear envelope pathological marker.

We computed a protection-associated repertoire score (0–3) representing the number of protection-associated phenomena per neuron. Among all patient neurons, TDP-43 depletion rates decreased as a function of protection-associated repertoire (Fig. 3b): 15.3% at a score of 0 (n = 10,069), 4.9% at a score of 1 (n = 2,655), 3.1% at a score of 2 (n = 509), and 2.3% at a score of 3 (n = 43; Kendall’s τ = −0.135, p = 1.3 × 10^− 56)^.

### Protection-associated phenomena cluster together within individual neurons

Pairwise analysis of all phenomenon combinations within patient nuclear TDP-43-preserved neurons revealed a striking block structure (Fig. 3c; note that the correlation matrix is computed across all disease neurons, whereas the odds ratios reported here are restricted to nuclear TDP-43-preserved neurons). Protection-associated phenomena co-occurred with each other at rates well above chance: PML nucleolar contacts with SUMO1 granules (OR = 3.3, p = 10^−40^) and with SUMO1 puncta (OR = 4.0, p = 10^− 49^). SUMO1 granules and puncta were near-independent (OR = 0.84, p = 0.054), possibly reflecting alternative morphological presentations of the same nucleolar SUMO1 response.

Protection-associated and vulnerability-associated phenomena rarely co-occurred within individual cells. Every protection-associated × vulnerability-associated pair showed an OR < 1, with most reaching statistical significance. The most frequent multi-phenomenon combinations were exclusively protection-associated (SUMO1 granules + PML nucleolar contacts, n = 195; SUMO1 puncta + PML nucleolar contacts, n = 180) or exclusively vulnerability-associated (PML membrane + macro-PML, n = 63) (Fig. 3d; counts in the panel are for all disease neurons, in which this combination totals 247). Mixed protection-associated-vulnerability-associated combinations were exceptionally rare (0.37% of nuclear TDP-43-preserved neurons; 43 of 11,593), consistent with two functionally segregated nuclear remodeling programs.

### Consistency across FTD genetic subtypes

The protection-associated and vulnerability-associated programs were largely shared across sporadic and *C9orf72*-associated FTLD-TDP. The protection-associated/vulnerability-associated classification held in both genotypes, and overall TDP-43 depletion burden did not differ between them (odds ratio 0.93, p = 0.82). A formal phenomenon × *C9orf72*-status interaction analysis identified a single robust difference: the association between PML nuclear membrane contacts and TDP-43 depletion was stronger in sporadic than in *C9orf72* disease (odds ratio approximately 17 in sporadic vs 6 in *C9orf72*; interaction p = 0.005–0.006 by cluster-robust logistic regression and generalized estimating equations, p < 0.001 by random-intercept mixed model, q = 0.03 after Benjamini–Hochberg correction, and significant in every leave-one-subject-out refit). Interaction terms for PML nucleolar contacts and macro-PML bodies were significant under some specifications but not others and were not robust to leave-one-subject-out resampling, so we do not interpret them. These programs therefore appear to represent broadly shared FTLD-TDP mechanisms, with the nuclear-envelope PML response more tightly coupled to TDP-43 depletion in sporadic disease.

## Discussion

In this study, we used quantitative multichannel immunofluorescence microscopy to identify two opposing remodeling programs within phase-separated nuclear compartments in FTLD-TDP. Three SUMO1- and PML-associated nucleolar phenomena followed inverted-U trajectories, becoming more frequent in patients but only in neurons with preserved nuclear TDP-43, consistent with a protective or compensatory response. PML redistribution to the nuclear envelope, showed a contrasting pattern in which prevalence increased in all patient neurons but especially those with nuclear TDP-43 depletion, suggesting a pathological change that precedes TDP-43 mislocalization. The findings nominate phase-separated nuclear PQC compartment status as one factor determining neuronal outcome in FTLD-TDP.

### Phase-separated nuclear PQC and neuronal protection.

PML nuclear bodies are phase-separated condensates assembled through SUMO–SIM interaction networks that create a scaffold for recruiting client proteins [7,28,47]. They function as nuclear PQC compartments, sequestering misfolded proteins and coordinating their processing through SUMOylation-ubiquitination cascades and p97/VCP-mediated disaggregation [19,22,27,38,45]. The nucleolus, another phase-separated structure, has similarly been implicated in protein sequestration under stress [9,10,16,29]. The protection-associated program we identified, SUMO1 nucleolar accumulation co-occurring with PML nucleolar contacts, may represent the convergence of these two PQC compartments in response to the broader proteotoxic stress that characterizes vulnerable neuronal populations in FTLD-TDP. Neurons exhibiting this nucleolar PQC response show markedly lower TDP-43 depletion rates, falling from 15.3% in neurons with none of these protection-associated phenomena to 2.3% in those with all three, suggesting that nucleolar PQC is associated with retention of nuclear TDP-43. Notably, the nucleolus is a site of quality control for diverse misfolded and aggregation-prone proteins. The protective response we observed may reflect a general nucleolar stress response to proteotoxic burden rather than a TDP-43-specific effect, and this question could be addressed in future studies. Rare patients with *C9orf72*-FTD show disabling FTD symptoms despite lacking extensive brain atrophy or TDP-43 aggregation at autopsy [18,51]. One such patient (Case 1 in [51]) was included in this study, and we noted with interest that this participant showed the highest proportion of neurons bearing the protection-associated nucleolar PQC features.

### SUMO1 nucleolar accumulation as a distinct pathway.

The SUMO1 nucleolar phenomena identified here, granules and puncta co-localizing with B23/nucleophosmin, are mechanistically distinct from the SUMO2/3-mediated PML body pathway characterized in cellular models of TDP-43 quality control [35,52,55]. Whereas SUMO2/3 chains mediate canonical PML body assembly and client recruitment in the nuclear interior, our findings implicate SUMO1 at the nucleolus, a different nuclear compartment with a different SUMO paralog. The strong co-occurrence of SUMO1 phenomena with PML nucleolar contacts suggests coordinated recruitment of PML-associated quality control machinery to nucleolar sites of SUMO1 accumulation. The SUMO1 nucleolar accumulation we observe in patient neurons with preserved nuclear TDP-43 is consistent with the SUMO-1 redistribution to nucleolar structures reported under proteasome inhibition, where quantitative proteomics identified 193 nucleolar SUMO-1 substrates including nucleophosmin/B23, the same marker that co-localizes with our SUMO1 granule phenomenon, as well as histones, ribosomal proteins, and RNA-binding proteins [36]. These observations suggest that the protection-associated nucleolar response we identified may reflect widespread engagement of this quality control pathway under disease-associated proteostatic stress [26,30,56]. The identity of the specific SUMOylated substrates accumulating at the nucleolus in patient neurons cannot be determined from morphological data alone and will require complementary approaches.

### PML redistribution to the nuclear envelope.

PML nuclear membrane contacts showed the most dramatic disease-associated enrichment of any phenomenon we observed (199-fold increase from control neurons to patient nuclear TDP-43-depleted neurons). Under normal conditions, PML bodies maintain their spherical condensate identity through the SUMO–SIM interaction network [7,28,47]. The mechanism driving PML redistribution to the nuclear envelope cannot be determined from cross-sectional, morphological data, and at least two non-exclusive possibilities warrant consideration. One possibility is a PML response to nuclear envelope stress or damage. PML nuclear bodies accumulate at the nuclear envelope in response to membrane curvature and elastic stress, and the PML-II isoform associates with the inner nuclear membrane, forming envelope-associated structures depleted of canonical PML body-associated proteins [37,42]; recent work further suggests such structures gather at sites of lamina weakening and impending rupture and disassemble once repair is initiated [23]. Because nuclear envelope and nucleocytoplasmic trafficking abnormalities have been implicated in TDP-43 mislocalization in ALS-FTD [13,57], a PML response to nuclear envelope stress could represent a change that precedes, rather than follows, nuclear TDP-43 depletion. Under this interpretation, the PML nuclear membrane contacts we observe would represent a potentially adaptive, surveillance-type response rather than a sign of PML body dysfunction. This interpretation aligns with the recent description of karyoptosis, a caspase-independent death pathway in which p38-mediated lamin B1 phosphorylation destabilizes the nuclear lamina in step with declining nuclear TDP-43 [12]. One limitation pertains. Our antibody is pan-PML, so we cannot establish PML-II specificity, and our pan-PML signal localizes to the nuclear envelope but does not resolve the fine patch morphology reported for these PML-II structures. The envelope-stress-PML link therefore remains a mechanistically consistent hypothesis, not a demonstrated mechanism.

The second possible explanation is that the PML redistribution to the nuclear envelope represents a downstream feature of a broader proteostatic stress response that somehow puts neurons at risk for TDP-43 mislocalization. The nuclear envelope repair vs. proteostatic stress response hypotheses are not mutually exclusive, and our cross-sectional data cannot distinguish between them. The proteostatic overload interpretation applies most directly to the enlarged Macro-PML bodies, which we consider next. Macro-PML bodies co-occurred strongly with nuclear membrane contacts (OR = 95.8) and with TDP-43 depletion (OR = 20.2), yet ~ 29% of Macro-PML-bearing neurons lacked membrane contacts, indicating that the two are related but not equivalent. Our cross-sectional, pan-PML data cannot establish whether they are a distinct state, a later stage of the same response, or the same structures at higher PML load. One candidate mechanism is impairment of the VCP/p97 segregase, which extracts ubiquitinated, SUMOylated clients from PML bodies for degradation. Mutations in VCP cause multisystem proteinopathy with frontotemporal dementia and FTLD-TDP, Type D [8,21,24,54]. Whether Macro-PML bodies reflect impairment of this step is a hypothesis we cannot test here, though the enlargement of PML foci could represent an overloaded state in which impaired clearance of defective ribosomal products coincides with disrupted PML condensate dynamics [4,38].

### Convergent evidence and a model of opposing nuclear PQC trajectories.

The strength of the protection-associated versus vulnerability-associated framework proposed here derives from the convergence of independent approaches. Cross-sectional analysis provides the initial classification based on neuronal population-level prevalence patterns. Co-occurrence analysis adds a single-cell dimension: protection-associated and vulnerability-associated phenomena segregate into different neurons (OR < 1 for all cross-class pairs), defining two mutually exclusive cellular programs. Integrating these findings, we propose a model in which the FTLD-TDP milieu elicits two competing neuronal responses. In the protection-associated trajectory, proteostatic stress, of the kind shown to redistribute SUMO1 to nucleolar structures under proteasome inhibition [36], triggers enhanced SUMO1 accumulation at the nucleolus with concurrent PML recruitment to the nucleolar periphery, reinforcing nucleolar PQC capacity. By managing the broader proteotoxic burden, this response may preserve the cellular conditions under which TDP-43 maintains its normal nuclear localization and function. In the vulnerability-associated trajectory, the PML condensate system is destabilized: PML bodies redistribute from the nuclear interior to the envelope, losing compartmentalized PQC function. The mutual exclusivity of these programs at the single-cell level suggests that once a neuron commits to one trajectory, it does not readily switch to the other.

### Limitations.

Imaging was performed as single-plane 2D acquisition, which may miss three-dimensional spatial relationships, particularly for nuclear envelope contacts. This study was cross-sectional; the prevalence analysis relies on comparing different neuronal populations as a proxy for temporal progression, but our study design cannot confirm temporality or causality. Because regions of interest were selected in part to enrich for nuclear TDP-43-depleted neurons, the overall proportion of nuclear TDP-43-depleted neurons reflects the sampled regions rather than an unbiased regional prevalence; because phenomena were scored per neuron while blinded to TDP-43 status, however, this sampling is not expected to bias the within-neuron associations between phenomena and TDP-43 status. Prevalence was necessarily measured in neurons surviving to autopsy. If nuclear TDP-43-depleted neurons are preferentially lost, the depleted neurons sampled here represent a surviving subset rather than the full population that passed through that state, and prevalence within this group is therefore conditioned on survival. This constraint applies to any single-timepoint post-mortem measurement and cannot be resolved without longitudinal observation. Analyses were restricted to a single brain region, anterior cingulate cortex, albeit one that is early-affected in bvFTD [49]. Despite these limitations, the convergence of two analytical approaches, the consistency across genetic subtypes, and the biological plausibility of the findings support the proposed framework.

### Future directions.

The molecular identity of the SUMOylated substrates accumulating at the nucleolus remains unknown and represents one important mechanistic question arising from this work. Laser-capture microdissection of neurons stratified by SUMO1 phenomena, coupled with proteomics, could elucidate the specific targets of this response. Whether SUMO1 nucleolar accumulation and SUMO2/3-mediated PML body quality control represent parallel, sequential, or competing pathways with distinct substrate specificities warrants investigation. Perhaps most intriguingly, if SUMO1 nucleolar accumulation represents a conserved neuronal response to proteotoxic stress rather than a TDP-43-specific phenomenon, it should be detectable in other proteinopathies where nuclear proteotoxic burden is significant, such as tauopathies [50] and polyglutamine repeat disorders [43,46,48]. Systematic examination of SUMO1 nucleolar status across neurodegenerative diseases could determine whether this phenomenon serves as a general marker of nuclear proteotoxic stress, potentially providing a morphological readout of proteostatic burden that is independent of the specific misfolded protein involved. Extending these analyses to other brain regions within FTD and to other TDP-43 proteinopathies, including limbic-predominant age-related TDP-43 encephalopathy (LATE) [3,25,40], would further test the generalizability and specificity of these findings.

## Conclusions

We identified two opposing remodeling programs within phase-separated nuclear PQC compartments in FTLD-TDP: (1) a protection-associated pattern in which the nucleolus accumulates SUMO1 and is surrounded by PML bodies and (2) a PML redistribution to the nuclear envelope that may precede and then escalate with TDP-43 pathobiology. These findings expand the known pre-TDP-43 depletion changes and provide a framework for understanding how the functional state of nuclear condensates influences neuronal vulnerability in FTD and ALS.

## Supplementary Material

Supplementary Files

This is a list of supplementary files associated with this preprint. Click to download.

- 02Table1.docx
- ESM1.docx

## Figures and Tables

**Figure 1:**
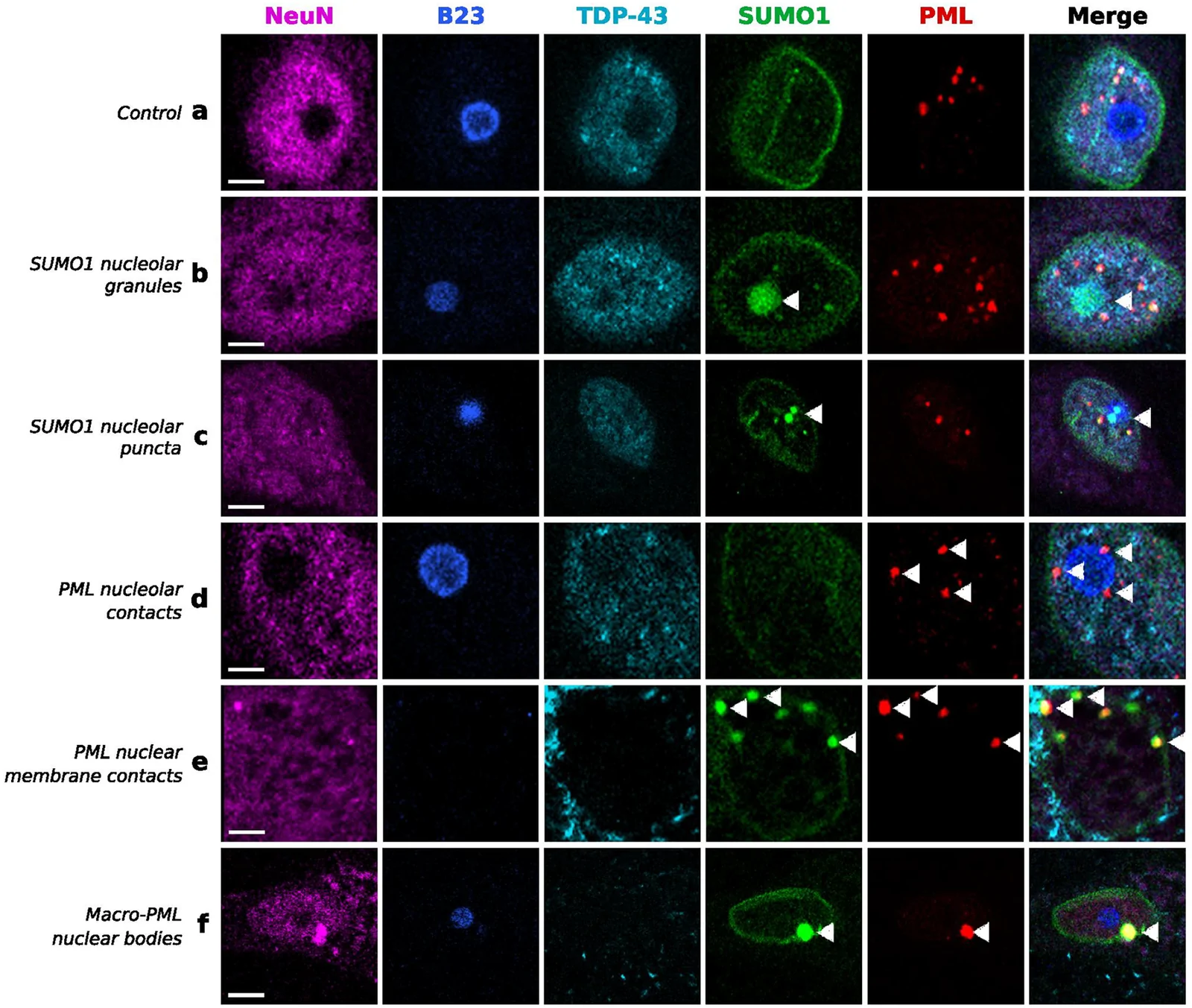
Five nuclear body phenomena and a control neuron in anterior cingulate cortex neurons resolved by five-color immunofluorescence. Each row shows one neuron in five single-channel images and a merge of all five channels. Columns (left to right): NeuN, B23/nucleophosmin (nucleolar marker), TDP-43, SUMO1, PML, and merge. a Neurologically normal control neuron. b SUMO1 nucleolar granules. c SUMO1 nucleolar puncta. d PML nucleolar contacts. e PML nuclear membrane contacts. f Macro-PML nuclear bodies. Arrowheads mark the scored feature; because the merge composites all five channels, co-localization is best assessed in the single-channel panels rather than in the merge. Images are single-plane confocal acquisitions (Leica SP8) with display intensities scaled linearly per channel using limits common to all panels; all panels share an identical 22.1 × 22.1 μm field at identical magnification; scale bars, 5 μm

**Figure 2:**
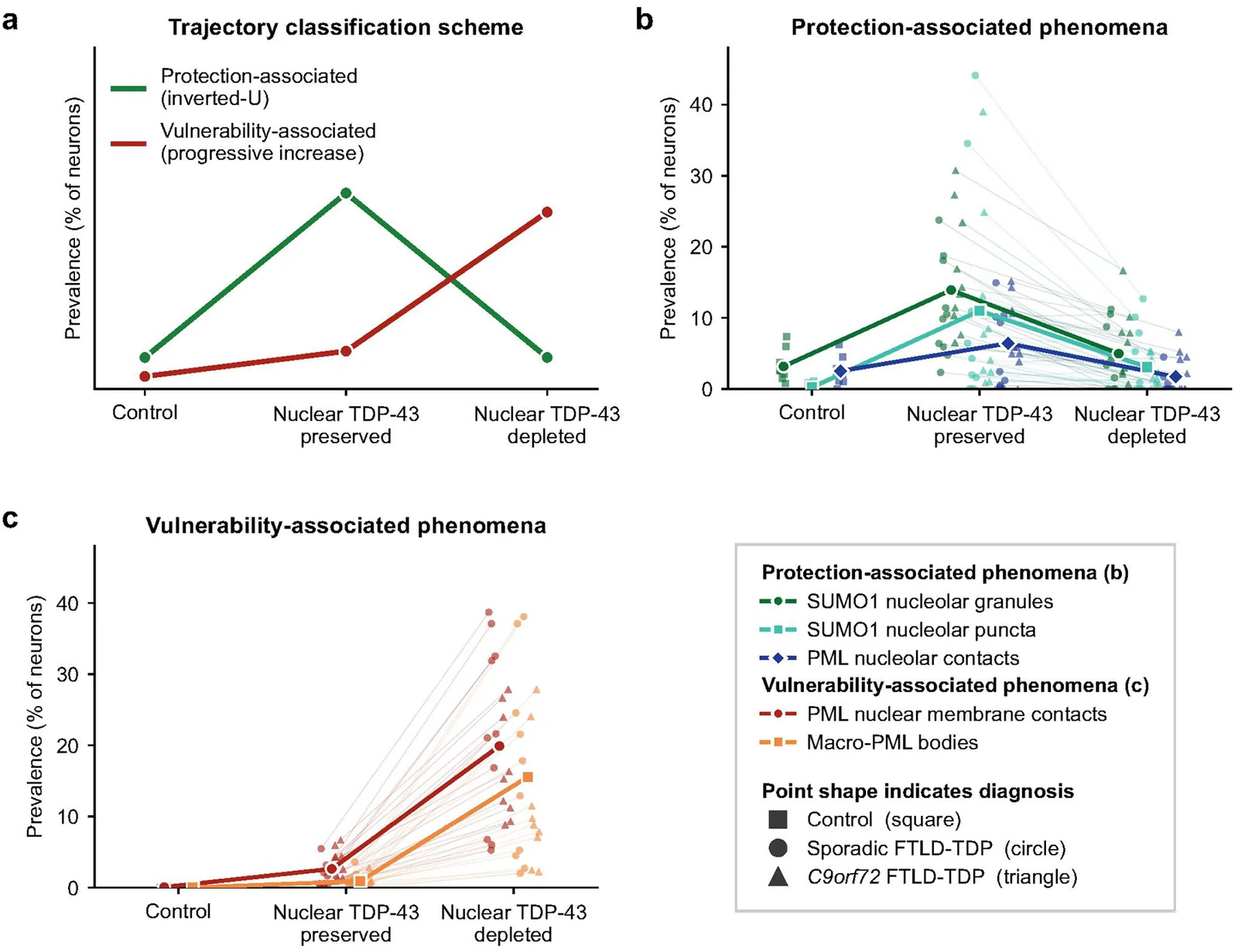
Cross-sectional prevalence analysis distinguishes protection-associated from vulnerability-associated phenomena. a Schematic of the trajectory classification scheme. Phenomena were classified as protection-associated when prevalence peaked in disease nuclear TDP-43-preserved (surviving) neurons and returned toward control levels in nuclear TDP-43-depleted neurons, an inverted-U, and as vulnerability-associated when prevalence increased progressively across the three populations, with the largest increment in nuclear TDP-43-depleted neurons. Segments are drawn between the three population values only and are not intended to interpolate intermediate states. b Protection-associated phenomena (SUMO1 nucleolar granules, SUMO1 nucleolar puncta, PML nucleolar contacts) follow inverted-U trajectories across control, disease nuclear TDP-43-preserved, and disease nuclear TDP-43-depleted neurons, peaking in nuclear TDP-43-preserved neurons and declining with TDP-43 depletion, consistent with compensatory responses that are lost when TDP-43 nuclear integrity fails. c Vulnerability-associated phenomena (PML nuclear membrane contacts and Macro-PML bodies) are near-absent in controls and increase progressively through TDP-43 depletion, reaching 19.9% and 15.6% of nuclear TDP-43-depleted neurons, respectively, consistent with escalating vulnerability-associated pathology. In b and c, each phenomenon is assigned its own color, used for both its trajectory line and its per-subject points, so that individual points can be attributed to the corresponding phenomenon. Large markers and lines show pooled prevalence; small points show per-subject prevalence, with point shape indicating diagnosis (squares, controls; circles, sporadic FTLD-TDP; triangles, *C9orf72*-associated FTLD-TDP). Controls are shown once and shared across both patient groups, since control neurons were not subdivided by TDP-43 status. Thin lines join the two values contributed by the same patient and do not represent change within neurons over time. Prevalence is expressed as a percentage of the neurons counted in the sampled fields, not of the original neuronal population.

**Figure 3:**
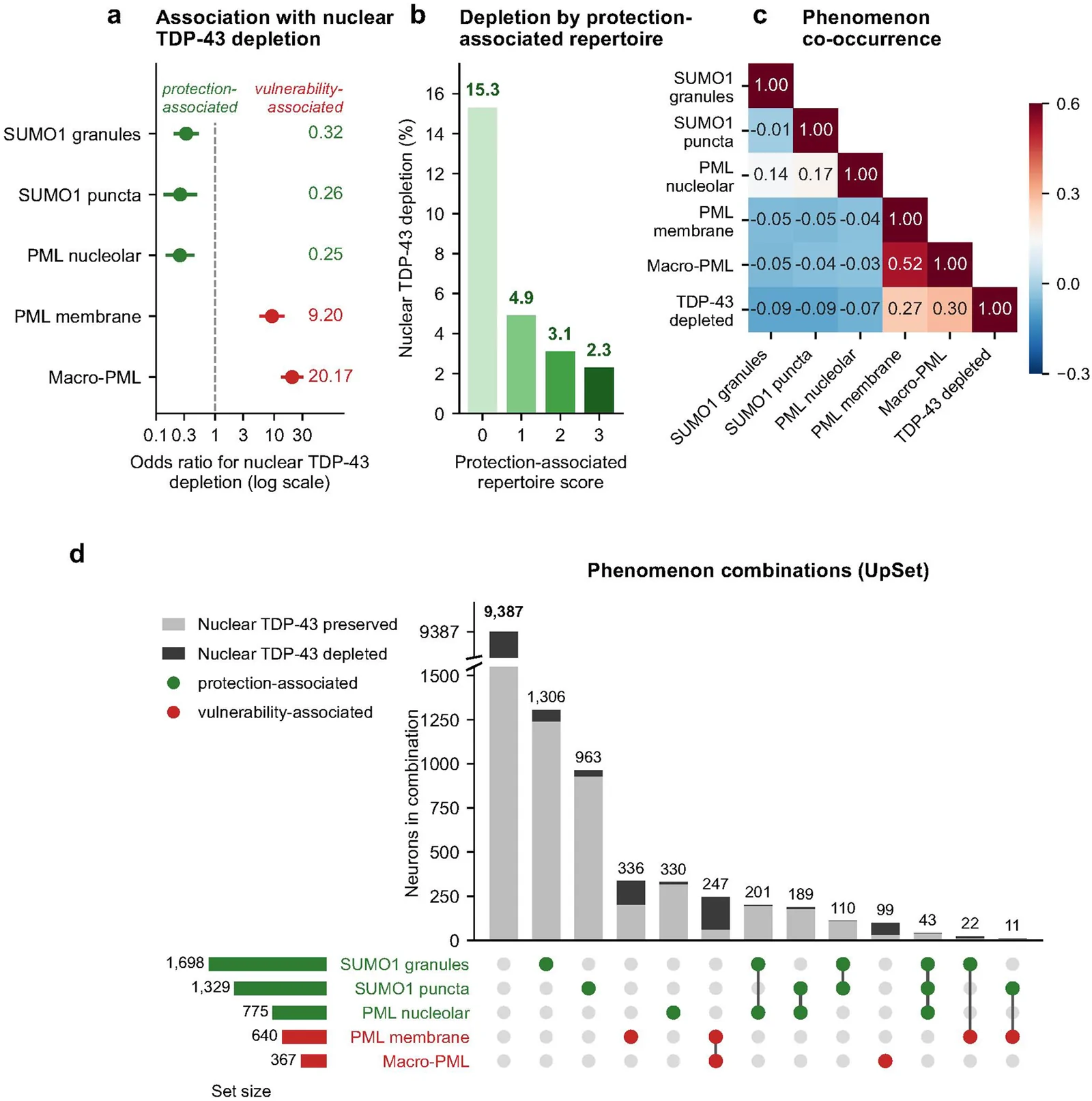
Protection-associated and vulnerability-associated phenomena show opposite associations with TDP-43 depletion. a Forest plot of the odds ratio for each phenomenon’s association with nuclear TDP-43 depletion across all disease neurons (log scale; whiskers, 95% CI; dashed line, OR = 1). The three protection-associated phenomena are under-represented in nuclear TDP-43-depleted neurons (OR < 1), whereas the two vulnerability-associated phenomena, PML nuclear membrane contacts and Macro-PML bodies, are strongly enriched (OR = 9.2 and 20.2). b Among disease neurons, the proportion retaining normal nuclear TDP-43 rises with the protection-associated repertoire score, the number of protection-associated phenomena present per neuron (0–3). Group sizes are n = 10,069, 2,655, 509 and 43 neurons for scores 0–3. c Pairwise φ-correlation matrix for the five phenomena and TDP-43 depletion across all disease neurons (lower triangle shown). The two vulnerability-associated phenomena correlate positively with each other and with TDP-43 depletion, whereas the protection-associated phenomena are weakly positively correlated or essentially uncorrelated among themselves (φ = −0.01 to 0.17) and negatively correlated with the vulnerability-associated phenomena and with depletion. d UpSet plot of phenomenon combinations across all disease neurons. Each column is one combination of phenomena, indicated by the filled dots below; bar height gives the number of neurons with that combination, with the stack split by TDP-43 status (light gray, nuclear TDP-43-preserved; dark gray, nuclear TDP-43-depleted). Dots are colored by phenomenon class (green, protection-associated; red, vulnerability-associated), and the leftmost column is the no-phenomenon state (9,387 neurons). Horizontal bars at left give the total number of neurons positive for each phenomenon (set size). Protection-associated combinations occur almost exclusively in nuclear TDP-43-preserved neurons; the vulnerability-associated combination (PML nuclear membrane contacts together with Macro-PML bodies) is enriched in nuclear TDP-43-depleted neurons; and mixed protection-associated–vulnerability-associated combinations are rare. Combinations occurring in fewer than 10 neurons are omitted for legibility (32 neurons in total), so the plotted columns sum to 13,244 of the 13,276 disease neurons.

**Table 1:** Case characteristics Twenty-nine subjects (10 sporadic FTLD-TDP, 10 C9orf72-associated FTLD-TDP, 9 neurologically normal controls) selected by consensus review of the UCSF Neurodegenerative Disease Brain Bank. Case identifiers are brain-bank accession numbers. Values are per case; group summaries give mean ± SD.

Case	Group	Sex	Age	Clinical diagnosis	Primary neuropathological diagnosis	C9orf72	PMI (h)	ADNC	Braak NFT stage	Thal phase	CERAD	LBD stage	CAA	Other neuropathological findings
P2143	Sporadic FTLD-TDP	F	54	bvFTD (R temp)	FTLD-TDP-B	—	16.7	Not	0	0	0	—	—	Arteriosclerosis
P2260	Sporadic FTLD-TDP	F	62	bvFTD-MND	FTLD-TDP-B, ALS	—	42.3	Not	I	4	2	—	Present	ARDC
P2325	Sporadic FTLD-TDP	M	66	FTD-ALS	FTLD-TDP-B, MND	—	12.1	Not	II	0	0	—	—	non specific tauopathy
P2331	Sporadic FTLD-TDP	F	65	bvFTD	FTLD-TDP-B	—	8.5	Not	0	0	0	—	—	AGD
P2374	Sporadic FTLD-TDP	M	72	bvFTD-MND	FTLD-TDP-B	—	6.7	Not	IV	0	0	—	—	AGD, diffuse; ADRC
P2434	Sporadic FTLD-TDP	F	67	ALS	ALS-TDP	—	5.3	Low	II	4	2	—	Present	Atherosclerosis
P2472	Sporadic FTLD-TDP	M	56	nfvPPA-ALS	FTLD-TDP-B, MND	—	12.9	Not	0	0	0	—	—	—
P2946	Sporadic FTLD-TDP	M	57	bvFTD-ALS	FTLD-TDP-B, MND	—	7.6	Not	0	0	0	—	—	Argyrophilic grain disease, limbic type; Atypical tauopathy, affecting limbic structure
P3169	Sporadic FTLD-TDP	F	65	ALS	ALS-TDP, FTLD-TDP-B	—	12.7	Not	I	0	0	—	—	FTLD-TDP-B; microinfarct aCC
P3270	Sporadic FTLD-TDP	M	76	bvFTD vs. sbvFTD	FTLD-TDP-B	—	10	Intermediate	IV	3	2	—	—	Arteriosclerosis
P2305	C9orf72 FTLD-TDP	M	68	bvFTD-MND	FTLD-TDP-B, MND	Expanded	7.5	Not	III	0	0	Limbic	—	ADRC
P2308	C9orf72 FTLD-TDP	M	71	svPPA (right)	FTLD-TDP-B, MND	Expanded	11.5	Intermediate	IV	2	2	—	—	—
P2377	C9orf72 FTLD-TDP	F	65	bfFTD	FTLD-TDP-U	Expanded	17.6	Low	III	1	0	—	—	Arteriosclerosis
P2437	C9orf72 FTLD-TDP	M	68	FTD-ALS	FTLD-TDP-U, MND	Expanded	9	Low	I	1	0	—	—	AGD, limbic
P2706	C9orf72 FTLD-TDP	F	45	bvFTD-ALS	FTLD-TDP-B, ALS	Expanded	15.5	Intermediate	I	1	0	—	—	PART
P2793	C9orf72 FTLD-TDP	F	76	bvFTD	FTLD-TDP-B, MND	Expanded	5.6	Low	I	3	2	—	—	AGD, limbic
P2845	C9orf72 FTLD-TDP	F	41	bvFTD	FTLD-TDP-B, ALS	Expanded	17.1	Low	0	2	0	—	—	Arteriolosclerosis
P3094	C9orf72 FTLD-TDP	F	52	bvFTD-MND	FTLD-TDP-B, MND-TDP	Expanded	8.6	Not	I	0	0	—	—	Arteriosclerosis
P3135	C9orf72 FTLD-TDP	F	53	bvFTD	FTLD-TDP-B	Expanded	24.3	Not	I	0	0	—	—	ALS-TDP; PART
P3139	C9orf72 FTLD-TDP	M	73	bvFTD	FTLD-TDP-U	Expanded	11.8	Low	III	1	0	—	Present	Hippocampal Sclerosis; AGD, limbic
P2394	Control	M	76	Control	—	—	8.2	Not	I	1	0	—	—	AGD, limbic
P2594	Control	F	86	Control	—	—	6.4	Not	II	0	0	—	—	AGD, limbic
P2921	Control	F	82	Control	—	—	9.6	Low	I	2	0	—	—	—
P2937	Control	F	60	Control	—	—	20.5	Not	0	3	0	—	—	—
P2942	Control	F	67	Control	—	—	19.4	Not	0	0	0	—	—	—
P3041	Control	F	78	Control	—	—	11.6	Not	I	1	0	—	—	—
P3045	Control	F	84	Control	—	—	7.7	Low	II	2	1	—	Present	Vascular brain injury, microinfarct, old
P3075	Control	M	72	Control	—	—	< 19	Not	II	2	0	—	—	—
P3267	Control	M	79	Control	—	—	8.8	Not	I	2	1	—	—	old microinfarcts, insula, motor cortex
**Sporadic FTLD-TDP summary**		**5M/5F**	**64.0 ± 7.0**				**13.5 ± 10.7**							
**C9orf72 FTLD-TDP summary**		**4M/6F**	**61.2 ± 12.4**				**12.8 ± 5.7**							
**Control summary**		**3M/6F**	**76.0 ± 8.4**				**11.5 ± 5.4**							

ADNC, Alzheimer disease neuropathologic change (National Institute on Aging–Alzheimer's Association); Braak NFT stage, neurofibrillary tangle stage; Thal phase, amyloid-β phase; CERAD, Consortium to Establish a Registry for Alzheimer's Disease neuritic plaque score; LBD, Lewy body disease; CAA, cerebral amyloid angiopathy; PMI, post-mortem interval; TDP-43 NCI, bvFTD, behavioural variant frontotemporal dementia; nfvPPA, non-fluent variant primary progressive aphasia; svPPA, semantic variant primary progressive aphasia; ALS, amyotrophic lateral sclerosis; MND, motor neuron disease; —, not applicable or not recorded.

Group comparisons: age, one-way ANOVA F(2,26) = 6.28, p = 0.006 (Kruskal–Wallis p = 0.009); post-mortem interval, F(2,25) = 0.14, p = 0.87; sex distribution, χ^2^ p = 0.76. Controls were older than both patient groups, as is typical for post-mortem control cohorts; the groups did not differ in sex or post-mortem interval.

Post-mortem interval for case P3075 is recorded only as < 19 h and is excluded from the control-group mean (n = 8 of 9).

Cohort composition. Patient cases are FTLD-TDP type B/U: 16 of 20 are type B and three C9orf72 cases (P2377, P2437, P3139) are type U; one sporadic case (P2434) carries a primary diagnosis of ALS-TDP. Clinical presentations include FTD-ALS, ALS, nfvPPA-ALS and svPPA in addition to bvFTD. The 9 controls carry no FTLD-TDP designation and are excluded from the subtype denominator.

## Data Availability

The per-neuron scoring data and the analysis code that generate all figures and statistics reported in this article are openly available in Zenodo at https://doi.org/10.5281/zenodo.22802755, under a Creative Commons Attribution 4.0 International licence. The deposit comprises the scoring table for all 18,742 neurons with a data dictionary, and the scripts that reproduce Figs. 2 and 3. Confocal image files are available from the corresponding author on reasonable request.
